# Cone-Beam Navigation Can Reduce the Radiation Exposure and Save Fusion Length-Dependent Operation Time in Comparison to Conventional Fluoroscopy in Pedicle-Screw-Based Lumbar Interbody Fusion

**DOI:** 10.3390/jpm12050736

**Published:** 2022-05-01

**Authors:** Sebastian Rohe, Patrick Strube, Alexander Hölzl, Sabrina Böhle, Timo Zippelius, Chris Lindemann

**Affiliations:** 1 Orthopedic Department Waldkliniken Eisenberg, Professorship of the University Hospital Jena, 07607 Eisenberg, Germany; s.rohe@waldkliniken-eisenberg.de (S.R.); p.strube@waldkliniken-eisenberg.de (P.S.); a.hoelzl@waldkliniken-eisenberg.de (A.H.); s.boehle@waldkliniken-eisenberg.de (S.B.); 2 Department of Orthopedic Surgery, University of Ulm, 89081 Ulm, Germany; information@rku.de

**Keywords:** cone-beam navigation, radiation dose, preparation time, resource utilization, operation time, learning curve

## Abstract

This study investigates the advantages and disadvantages of cone-beam-based navigated standardized posterior lumbar interbody fusion surgery (PLIF), regarding the radiation exposure and perioperative time management, compared to the use of fluoroscopy. Patients treated receiving an elective one- to three-level PLIF were retrospectively enrolled in the study. The surgery time, preparation time, operation room time, and effective dose (mSv) were analyzed for comparison of the radiation exposure and time consumption between cone-beam and fluoroscopy; Results: 214 patients were included (108 cone-beam navigated, and 106 traditional fluoroscopies). Using cone-beam navigation, reductions in the effective dose (2.23 ± 1.96 mSv vs. 3.39 ± 2.32 mSv, *p* = 0.002) and mean surgery time of 30 min (143.62 ± 43.87 min vs. 171.10 ± 48.91 min, *p* < 0.001) were demonstrated, which leveled out the extended preparation time of 7–8 min (37.25 ± 9.99 min vs. 29.65 ± 7.69 min, *p* < 0.001). These effects were fusion length dependent and demonstrated additional benefits in multisegmental surgeries. The cone-beam navigation system led to a reduction in the perioperative time requirements and radiation exposure. Furthermore, the controversially discussed longer preparation time when using cone-beam navigation was amortized by a shortened surgery time, especially in multilevel surgery.

## 1. Introduction

Lumbar spinal fusion is a standardized surgery that is used for degenerated spine segments that were previously treated with conservative therapy that had failed, and lumbar spinal fusion consists of decompression, mechanical stabilization, and alignment reconstruction with screws and rods together with a bone graft-based induction of a bone bridge [1,2,3]. In recent years, many innovations in intraoperative imaging have been established in spinal surgery. Cone-beam navigated screw implantation has thus far shown excellent results in terms of the accuracy and avoidance of complications [1,2,4]. In addition to the increased accuracy of implant placement [2,4,5], lower radiation exposures to the patient and staff are a potential advantage [6,7], especially when using low-dose protocols. As for disadvantages of using this kind of navigation, high acquisition costs and a longer preparation time needed before the start of the surgery have been mentioned. The radiation exposure of the patient using cone-beam navigation has already been studied several times, but the results are controversial. Currently, there is a lack of data on cone-beam navigation. and no studies have been performed comparing it clinically to conventional fluoroscopy while using a uniform surgical technique. Furthermore, the fusion length dependency and its advantages/disadvantages regarding the preparation and surgical time as well as the radiation exposure have not been sufficiently addressed thus far.

Therefore, the aim of the present study was to investigate the advantages and disadvantages of cone-beam-based navigated standardized fusion surgery of a degenerated lumbar spine compared to the use of fluoroscopy regarding the radiation exposure and perioperative time management. Our hypotheses were that the use of cone-beam navigation leads to reduced radiation exposure, a prolonged preparation time, and a decreased surgical time and that these effects are fusion length dependent. Our secondary aim was to investigate the effects of the learning curve on the preparation time and radiation dose.

## 2. Materials and Methods

### 2.1. Study Design, Patients and Groups

In this retrospective cohort study, patients who underwent elective traditional an open surgical approach-based single-to-multilevel posterior lumbar interbody fusion (PLIF) and transpedicular stabilization with pedicle screws because of symptomatic lumbar segment degeneration with/without spinal stenosis in our hospital between February 2016 and February 2018 were included. All patients were unsuccessfully conservatively treated before surgery for a period of at least six months. Patients with a degenerative spondylolisthesis up to a Meyerding grade of I and mild de novo scoliosis up to 20 °Cobb were not excluded. The PLIF and bilateral pedicle screw-based stabilization had to be performed in every segment that was operated on.

Patients with previous surgeries on the lumbar spine, emergency interventions, and patients younger than 18 years were not included. The study was approved by the institutional review board/Ethics Committee (RNr. 2018-1129-Daten).

**Figure 1:**
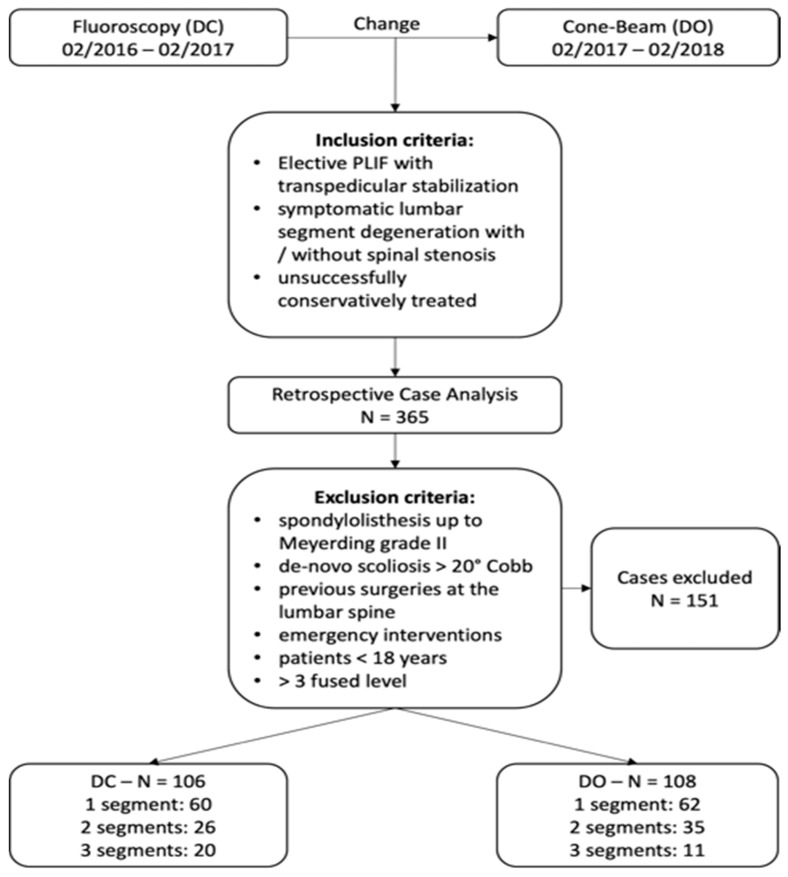
Patient recruitment flow chart.

The time frame of the study was set to the year before and the year after the standard intraoperative imaging technique had been changed from fluoroscopy to cone-beam-based navigation at the study site in February 2017. After the patients were selected (Figure 1), they were distributed into two groups according to the intraoperative imaging device that was utilized (DO: cone-beam-based navigation using O-ArmTM 2 and StealthstationTM 7, Medtronic, Minneapolis, MN, USA; or DC: fluoroscopy-based image guidance using C-Arm, Siemens Healthineers, Erlangen, Germany). To evaluate the patient’s safety, cases of documented revision surgeries for mispositioned pedicle screws and cases of intraoperative dural tears were collected in both groups.

### 2.2. Surgical Procedure

After general anesthesia, the patients were positioned in the prone position on a radiolucent carbon table in the operating room. The skin incision length was marked under fluoroscopic control using a group-specific device. After sterile draping of the patient and the devices, a posterior median traditional open approach was used, and the ROI of the lumbar spine was prepared. After implantation of the screws, decompression was performed in both groups via a flavectomy, a partial facetectomy and a caudal laminotomy. The discs were removed, and the cartilage was cleaned from the endplates using shavers, curettes and rongeur. The collected bone was morselized in a bone mill, and the bone was placed intervertebrally with press-fit sized morselized bone filled cages. After the screw-rod connection and the alignment correction as well as decortication of the remaining laminae and spinous processes, the remaining morselized bone was attached posteriorly for additional posterior spondylodesis induction. The incision was closed after irrigation and after the placement of a subfascial drainage, and a band-aid was applied under sterile conditions.

### 2.3. Radiation Exposure

In the DO group, the cone-beam device was positioned without sterility at the region of interest (ROI) and the anterior-posterior (a.p.), lateral view and parking position were set under the device’s fluoroscopic control. The aperture was employed to further focus on the surgical region during both scanning and fluoroscopic image acquisition. Subsequently, before scanning, the surgeon had to determine two settings: the patient thickness based on the patient’s BMI (small, medium, large, extralarge) and the dosage (low dose [LD], standard dose [SD], or high dose [HD]). After sterile draping and the preparation of the lumbar spine, the navigation reference was fixed on the most caudal spinous process, and a cone-beam-based scan was performed, while the staff left the operating room for radiation shielding. The acquired images were transferred to the optical navigation system for opto-navigation-assisted pedicle screw placement. The entire process of pedicle screw insertion was performed under navigation and without accompanying imaging. After the placement of all the screws, the position was checked with an a.p. and a lateral view of the device’s fluoroscopy. Only in uncertain situations where there was a possible displacement, a second scanning of the screws was performed with the cone-beam device. The cages were placed and navigated as well. After the screw-rod connection and, if necessary, repositioning, final fluoroscopic images were acquired in the a.p. and lateral position.

Similarly, in the DC group, the marking of the skin incision was performed using a.p. and lateral images with fluoroscopy. Usually, the aperture was employed to further focus on the surgical region in both devices. In the DC group, a fluoroscope was placed for a true lateral view of the ROI of the lumbar spine. The insertion of the screws was controlled during the insertion process for every screw. Following screw placement, an a.p. fluoroscopic control of the implant placement was performed. After placement of the cages, the position of the cages was checked fluoroscopically in two planes. Similar to the DO group, after the screw-rod connection, final fluoroscopy images were acquired in the a.p. and lateral position. During the entire period of fluoroscopy, the surgeons and staff wore lead aprons for radiation protection.

### 2.4. Quantification and Comparison of the Radiation Dose

The patient data were retrospectively collected from the electronic patient data of the HIS Orbis (Agfa Medical Care). The age, sex, body mass index (BMI), date of surgery, consecutive number of surgeries over time, the number of fused segments (1, 2, 3), dose area product (DAP) for patients in both of the groups and the dose length product (DLP) for the DO group, as well as the X-ray device used, surgery time, and preparation time, were determined.

The effective radiation dose E was determined by the International Commission on Radiological Protection system, which represents the tissue-weighted sum of the equivalent doses in all specified tissues and organs of the body [8]. A practical method to estimate the effective dose from the dose length product (DLP) by CT scan or DAP by fluoroscopy was performed by multiplying an established conversion factor by the radiation product [9]. The equivalent or effective dose (E in mSV) was calculated using the formula E = eDAP * DAP in the DC group, whereas 3 * 10^−4^ mSv/mGy*cm^2^ was assumed for eDAP, and E = DLP * f in the DO group. However, f = 0.0073 mSv/mGy*cm was assumed for men, and f = 0.01 mSv/mGy*cm was assumed for women [9].

### 2.5. Statistical Analysis

The statistical analysis was performed with IBM SPSS 28 (IBM, Armonk, NY, USA). The normal distribution of continuous variables was checked with the Kolmogorov–Smirnov test before performing parametric testing. The baseline data were compared using a 2-sided t-test or Chi-squared/Fisher’s exact test. The group comparisons were performed using a general linear model with post hoc Bonferroni tests. The level of significance was set to *p* < 0.05. The mean radiation dose E, surgical time, and preparation time were compared between the groups using the number of fused levels as cofactors. Based on the visualization of the preparation time over the case number, a cutoff was determined in the DO group to define the end of the learning curve. All of the parameters were compared again, excluding the cases during the learning curve from the DO group, to receive a routine clinical picture of the comparison.

## 3. Results

### 3.1. Demographics

A total of 214 patients were included in the analysis (Figure 1). The baseline characteristics, except sex, did not differ significantly between the groups (Table 1).

### 3.2. Surgical Data

A total of 122 patients received a 1-level fusion, 61 had a 2-level fusion, and 31 patients had a 3-level fusion (Table 1). The surgery times and radiation doses are shown in Table 2. Using cone-beam technology, the mean surgery time was 27.48 min shorter, and the mean preparation time was 7.6 min longer compared to conventional imaging. The effective radiation dose E was always lower in the DO group than in the DC group, with an average dose reduction of 1.16 mSv. In the DO group, low-dose protocols were used in 75 cases, standard-dose protocols in 33 cases and high-dose protocols in one case.

**Figure 2:**
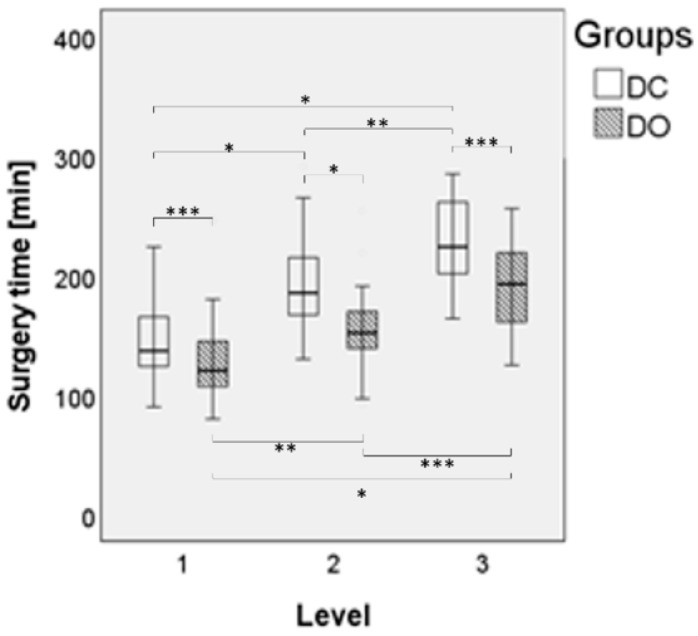
Boxplot for the surgery time depending on the number of fused levels, whisker with single standard deviation, * *p* ≤ 0.001, ** *p* ≤ 0.01, *** *p* ≤ 0.05.

**Figure 3:**
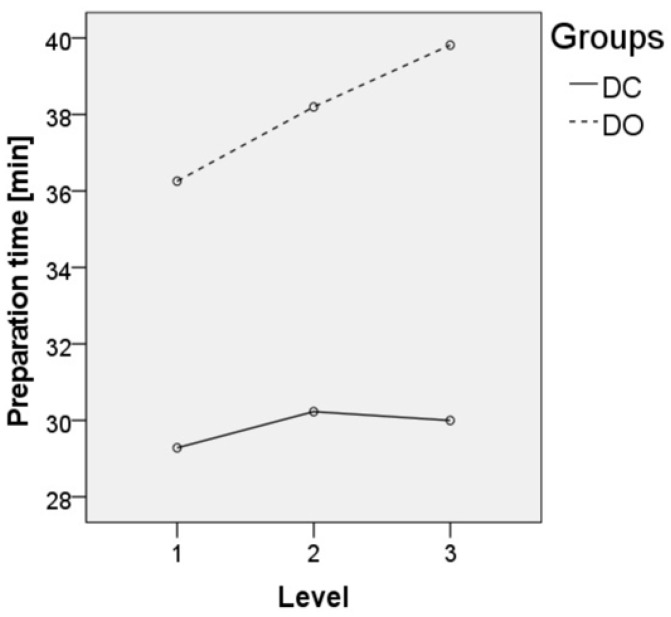
The preparation time in minutes depending on the number of fused levels.

**Figure 4:**
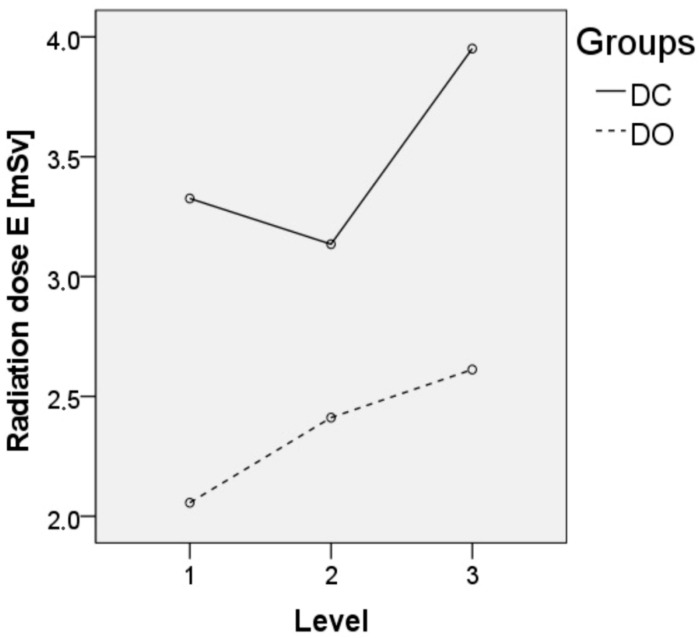
The radiation dose E in mSv depending on the number of fused levels.

Regarding the comparison of the imaging device, there was a significant effect on the surgery time (*p* < 0.001, Figure 2), preparation time (*p* < 0.001, Figure 3) and effective radiation dose (*p* = 0.002, Figure 4) between both groups (Table 2). Regarding the comparison of the number of fused vertebral levels, there was a significant effect on the surgery time (*p* < 0.001), which increased with a level extension, while the preparation time (*p* = 0.391) and effective radiation dose (*p* = 0.421) were not affected. A post hoc analysis showed a significant difference in the surgery time, which depended on the number of fused levels (*p* < 0.001) in both groups, with a longer time noted when there was a higher number of fused levels. Furthermore, the effective radiation dose, which depended on the device and the number of fused levels, showed no significant difference (*p* = 0.696). Regarding the patient’s safety, the revision rates due to pedicle screw malposition were comparable between the groups (6% in the DC group vs. 1% in the DO group, *p* = 0.118). The rates of intraoperative dural tears were also comparable between the groups (3% in the DC group vs. 2% in the DO group, *p* = 0.983). No major postoperative neurological complications could be observed in the presented study.

### 3.3. Learning Curve

**Figure 5:**
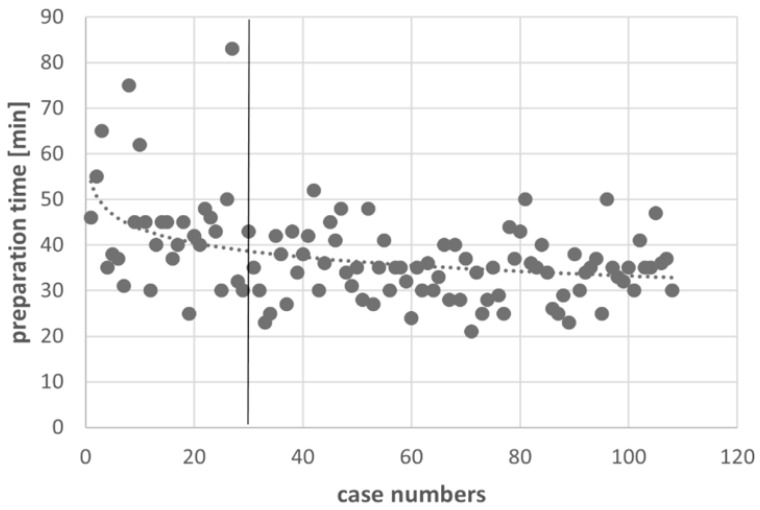
Learning curve utilizing cone-beam navigation showing the preparation time over the case numbers.

**Figure 6:**
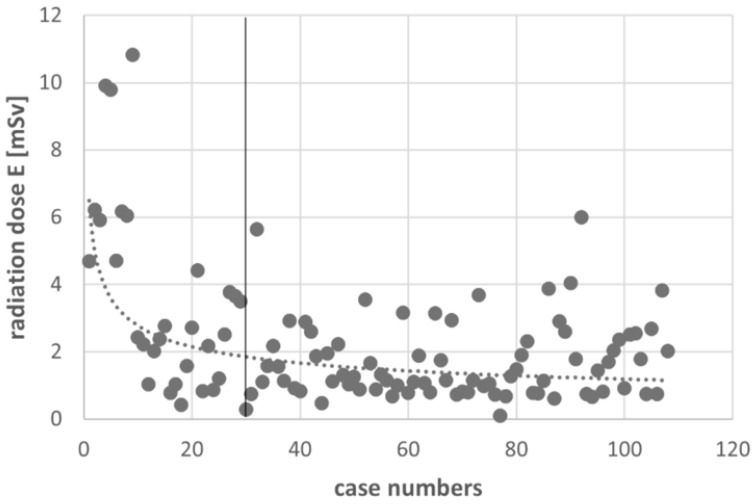
Learning curve utilizing cone-beam navigation showing the radiation dose E in mSv over the case numbers.

Figure 5 shows the course of the preparation time of the cone-beam navigation over the observation period. Initially, there was a spread of longer preparation times, which approached an approximate limit of 35 min after 30 surgeries were performed. After calculating the preparation times, surgery time and radiation dose E and after excluding the first 30 cases of the learning curve in the DO group, the mean difference in the preparation times between cone-beam navigation and fluoroscopy dropped to 5 min, and the mean E difference reached a higher significance (EDO decreased to 1.8 mSv, *p* < 0.001). Additionally, the surgery time decreased by approximately 5 min (Table 2, Figure 5 and Figure 6).

## 4. Discussion

The present study’s aim was to investigate the advantages and disadvantages of cone-beam-based navigated standardized fusion surgery of the degenerated lumbar spine compared to the use of fluoroscopy, and this study evaluated the radiation exposure and perioperative time management. Regarding our primary hypothesis, a reduction in the radiation dose to the patient by using cone-beam navigation could be demonstrated. Furthermore, a significant reduction of 30 min in the operation time by using cone-beam navigation, which leveled out the extended preparation time of 7–8 min, has been shown. These effects were fusion length dependent, and they demonstrated additional benefits in multisegmental surgeries.

Intraoperative image utilization is mandatory for spinal surgery. Therefore, it is important that our study confirm the reported radiation dose saving effect by using cone-beam navigation [10,11,12,13]. In the present study, the observed difference in the radiation dose can be explained by the different utilization of X-rays in both groups. In the DC group, every screw placement was accompanied by a fluoroscopic validation of the screw position. Therefore, the radiation dose was multiplied by the number of screws. In the DO group, only an initial scan was performed without an intraoperative validation of the screw positioning by using an X-ray. Thus, the radiation dose per screw had decreased with the number of implanted screws. Consequently, the overall radiation dose in the DO group had also decreased with increasing fusion levels. However, the results are in contrast with those of previous studies. Chang et al. reported an increased radiation dose in short-level fusions (≤6 screws) utilizing cone-beam navigation [10]. Nevertheless, the beneficial effect of cone-beam navigation on the radiation dose during multilevel fusions was also observed, while the significance of the difference in the dose was lost in long-level fusion surgery (>6 screws). In contrast, Mendelssohn et al. reported 2.77 times higher radiation exposure to the patient in cone-beam navigated surgery (first generation O-Arm, Medtronic) than in conventional fluoroscopy [12], and Kaminski et al. reported a higher radiation dose utilizing c-arm guided navigation using a conventional posterior approach (76.1 vs. 59.3 Gy/cm^2^) but without statistical significance (*p* = 0.166) [6]. A possible explanation of the different results of the present and previous studies could be that the radiation dose in cone-beam navigated spine surgery is dependent on several factors, i.e., the acquisition protocol (LD, HD) of the imaging device, the number of performed scans, the patients’ BMI, the surgical technique, and the surgery time. Since the patient’s habitus is a limiting factor for reducing the radiation dose while maintaining acceptable image quality, BMI should be taken into consideration to evaluate the radiation dose in cone-beam navigated, respectively, fluoroscopic guided spine surgery. Our study presents an overweight patient population with a mean BMI of 29 kg/m^2^. However, the mean BMI in the presented study was comparable between both groups.

In addition, other studies have also found reductions in the radiation dosage to the surgical team of 92% in cadaver studies [13], and 60% in clinical studies during navigated spinal fusion surgeries [11], especially when using minimally invasive approaches [14,15,16]. However, this comparison was not part of the present study.

In the present study, the radiation dose was 2.23 ± 1.96 mSV in cone-beam navigation compared to 3.39 ± 2.32 mSV in conventional fluoroscopy. A better way to understand the dose magnitude is to compare the doses with natural background radiation, which is 3.1 mSv per year for US citizens [17]. The mean effective dose for a lumbar spine CT is 7.5 mSv to 10 mSv [18]; a severely injured trauma patient receives an average of 22.7 mSv from the CT scans that are performed in the emergency department [19]. According to the 2007 recommendations of the International Commission on Radiological Protection, the adjusted nominal risk coefficient for cancer is 5.5 * 10^−2^ Sv^−1^ [8]. In conclusion, the cancer risk associated with the use of cone-beam navigation is present but is low compared to other medical procedures.

Our study confirms the known lengthening of the preparation time using cone-beam navigation. The overall surgery time in our study group was shorter using navigation, and it decreased further with an increasing number of implanted screws/fused segments. Previous studies that have considered the operating room time (preparation and surgery) reported no statistically significant difference between cone-beam navigated and fluoroscopically guided spinal fusion surgery [2,5,20]. Furthermore, the present study showed a prolonged preparation time in cone-beam navigation compared to fluoroscopy. However, based on the reduced surgical operation time, a positive effect on the overall operating room time was demonstrated. The present study confirms the results from Balling and Ryang [21,22], who also reported a decrease in the mean preparation time and mean radiation dose using cone-beam navigation or a 3D-c-arm. In our study, there were strong outliers at the beginning of the study due to the longer preparation times. However, with an increasing number of surgeries, the preparation time became shorter, which was approximately 35 min after 30 operations. In this context, the preparation time could be reduced by establishing a standardized workflow with positioning and sterile draping protocols, the use of positioning aids and the routine handling of the cone-beam device. Furthermore, the graphical analysis of the mean radiation dose curve showed a convergence of the curve toward a limit of approximately 1.8 mSv depending on the number of operations performed, with initially more frequent and higher peaks than in the further course due to a reduction of preoperative fluoroscopic positioning scans, the standardized use of low dose protocols and the waiving of the final scan by using only fluoroscopy in 2 planes.

Our study is not without limitations, and one limitation is its retrospective study design. First, the case numbers in both groups were different when based on the lumbar fusion levels, which may have an impact on the results. Second, the surgeries were performed by three orthopedic spinal surgeons with high but different expertise, and their different utilization of the C-Arm could influence the radiation dose. Consequently, the results could be different when the surgery is performed by a single physician or when different C-arm settings are used. Third, the O-arm was newly acquired, and the first surgeries showed a learning curve in terms of preparation and surgery times. Therefore, this led to a bias in the mean results but was considered in the post hoc analysis. Fourth, patients who underwent dorsal fusion surgery did not routinely receive postoperative computed tomography scans. Therefore, we are not able to draw conclusions concerning verification and assessment of pedicle screw accuracy. However, due to the very low clinically important screw malposition in the presented study, an additional computed tomography scan would only result in additional radiation exposure. Last, there might be some bias in the effective dose estimation. Theoretically, the effective dose was the tissue-weighted sum of the doses. If tissues were partially exposed to radiation, the estimation could have bias. However, even though the effective dose estimation is not a perfect measurement, it is still the best way to quantify the radiation exposure for the comparison and correlation with the epidemiological data for cancer risk estimation.

## 5. Conclusions

Our study demonstrated the successful inauguration of a cone-beam navigation system with a reduction in operating room time and radiation exposure. Furthermore, the controversially discussed longer preparation time using cone-beam navigation was confirmed but was amortized by a shortened surgery time, especially in multilevel surgery. This could lead to a reduction in the resource utilization of the operating room and a decrease in the radiation exposure to the operating room staff.

## Figures and Tables

**Table 1:** Baseline characteristics.

	Total	DC Group	DO Group	*p*-Value *
Age [y] ± SD	61.7 ± 12.2	60.8 ± 10.3	62.8 ± 13.9	0.291
Sex f:m, n	123:91	53:53	70:38	0.028
Mean BMI [kg/m^2^] ± [95%-CI]	29.4 ± 5.8 [28.6–30.1]	29.4 ± 6.4 [28.2–30.6]	29.3 ± 5.1 [28.4–30.3]	0.911
Number of patients	214	106	108	
1-level-fusion	122	60	62	0.986
2-level-fusion	61	26	35	0.202
3-level-fusion	31	20	11	0.071

* Analyzed with Student’s *t*-test for continuous variables and X2 for categorical variables. BMI: Body-mass-index; y: years; f: female; m: male; CI: confidence interval; DC: fluoroscopy-based image guidance; DO: cone-beam-based navigation; SD: single standard deviation.

**Table 2:** Operation time and radiation dose.

	DC Group	DO Group (Complete)	*p*-Value *	DO Group (Learning Excluded)	*p*-Value *
Surgery time [min] **	171.10 ± 48.91	143.62 ± 43.87	<0.001	138.51 ± 36.03	<0.001
Surgery time 1-level [min] **	144.10 ± 30.73	128.66 ± 42.05		122.43 ± 22.63	
Surgery time 2-level [min] **	190.88 ± 47.16	154.77 ± 31.70		152.62 ± 34.31	
Surgery time 3-level [min] **	226.40 ± 36.10	192.45 ± 44.11		187.14 ± 48.49	
Preparation time [min] **	29.65 ± 7.69	37.25 ± 09.99	<0.001	34.68 ± 6.86	0.001
Preparation time 1-level [min] **	29.28 ± 7.54	36.26 ± 07.92		34.98 ± 6.60	
Preparation time 2-level [min] **	30.23 ± 8.85	38.20 ± 11.69		34.38 ± 6.87	
Preparation time 3-level [min] **	30.00 ± 6.83	39.82 ± 14.28		33.86 ± 9.24	
Effective dose [mSv] **	3.39 ± 2.32	2.23 ± 1.96	0.002	1.76 ± 1.13	<0.001
Effective dose 1-level [mSv] **	3.33 ± 2.79	2.06 ± 1.97		1.64 ± 1.07	
Effective dose 2-level [mSv] **	3.14 ± 1.50	2.41 ± 2.08		1.90 ± 1.28	
Effective dose 3-level [mSv] **	3.95 ± 1.52	2.61 ± 1.51		2.01 ± 0.88	

* Student’s *t*-test; ** mean ± single standard deviation. DC: fluoroscopy-based image guidance; DO: cone-beam-based navigation.

## Data Availability

Not applicable.
